# Early clinical experience of radiotherapy of prostate cancer with volumetric modulated arc therapy

**DOI:** 10.1186/1748-717X-5-54

**Published:** 2010-06-16

**Authors:** Gianfranco A Pesce, Alessandro Clivio, Luca Cozzi, Giorgia Nicolini, Antonella Richetti, Emanuela Salati, Mariacarla Valli, Eugenio Vanetti, Antonella Fogliata

**Affiliations:** 1 Oncology Institute of Southern Switzerland, Radiation-Oncology Dept, Bellinzona, Switzerland; 2 Oncology Institute of Southern Switzerland, Medical Physics Unit, Bellinzona, Switzerland

## Abstract

**Background:**

To report about initial clinical experience in radiation treatment of carcinoma of prostate with volumetric modulated arcs with the RapidArc (RA) technology.

**Methods:**

Forty-five patients with a median age of 72 ± 3, affected by prostate carcinoma (T1c: 22 patients, T2a-b: 17 patients, T3a-b: 6 patients. N0: 43 patients, N1-Nx: 2 patients, all M0), with initial PSA of 10.0 ± 3.0 ng/mL, were treated with RapidArc in a feasibility study. All patients were treated with single arc using 6MV photons. Dose prescription ranged between 76 (7 patients) and 78 Gy (38 patients) in 2Gy/fraction. Plan quality was assessed by means of Dose Volume Histogram (DVH) analysis. Technical parameters of arcs and pre-treatment quality assurance results (Gamma Agreement Index, GAI) are reported to describe delivery features. Early toxicity was scored (according to the Common Terminology Criteria of Adverse Effects scale, CTCAE, scale) at the end of treatment together with biochemical outcome (PSA).

**Results:**

From DVH data, target coverage was fulfilling planning objectives: V_95% _was in average higher than 98% and V_107%_~0.0% (D_2%_~104.0% in average). Homogeneity D_5%_-D_95% _ranged between 6.2 ± 1.0% to 6.7 ± 1.3%. For rectum, all planning objectives were largely met (e.g. V_70Gy _= 10.7 ± 5.5% against an objective of < 25%) similarly for bladder (e.g. D_2% _= 79.4 ± 1.2Gy against an objective of 80.0Gy). Maximum dose to femurs was D_2% _= 36.7 ± 5.4Gy against an objective of 47Gy. Monitor Units resulted: MU/Gy = 239 ± 37. Average beam on time was 1.24 ± 0.0 minutes. Pre-treatment GAI resulted in 98.1 ± 1.1%. Clinical data were recorded as PSA at 6 weeks after RT, with median values of 0.4 ± 0.4 ng/mL. Concerning acute toxicity, no patient showed grade 2-3 rectal toxicity; 5/42 (12%) patients experienced grade 2 dysuria; 18/41 (44%) patients preserved complete or partial erectile function.

**Conclusion:**

RapidArc proved to be a safe, qualitative and advantageous treatment modality for prostate cancer.

## Background

In Switzerland an increasing incidence of prostate adenocarcinoma was observed in the last 10 years, with 5668 new cases/year, attaining to the 29.6% of all male malignancies in 2006, and an yearly average mortality of 1292 patients between 2003 and 2006 over a population of about 7.4 million inhabitants [1]. In our region (of about 320'000 inhabitants) the incidence of prostate adenocarcinoma is also increasing with average 170 new cases/year between 1996 and 2007 (mortality 47 patients/year, between 1996 and 2005). A large portion of those patients are treated by radiotherapy.

A proper planning policy, which allows to spare the healthy tissue and at the same time ensure high cure rate, is of particular importance due to the rate of curability of this tumour and long survival of the patients. In this respect new, highly conformal treatments have been tested in the last years.

Volumetric Modulated Arc Therapy (VMAT), based on the original investigation of K. Otto [2] has been recently introduced in clinical practice in several institutes after an intensive validation at planning level, compared to IMRT or other approaches. RapidArc (RA), the Varian solution of VMAT, is implemented as the Progressive Resolution Optimisation (PRO) algorithm in the Eclipse planning system by Varian Medical System (Palo Alto, California, USA). The optimisation process is based on an iterative inverse planning process aiming to simultaneously optimise the instantaneous multi leaf collimator (MLC) positions, the dose rate, and the gantry rotation speed to achieve the desired dose distribution.

Pre-clinical validation of RapidArc was addressed in a series of studies including brain tumours, head and neck, anal canal, cervix uteri cancer and other indications [3-9]. The potential role of RA in the treatment of prostate cancer has been investigated by the Danish group of Rigs Hospitalet [6], by the Vancouver group lead by K. Otto [10,11], by the group of Duke University [12] and by the group of Memorial Sloan Kettering [13].

At our institute, until end of January 2010, more than 250 patients have been treated with RapidArc for a variety of indications. Among these, 117 received RapidArc treatment as part of their multidisciplinary management of prostate adenocarcinoma.

Of these, forty-five, irradiated without inclusion of the pelvic nodes, were included in the present study based on the risk class. After a short transition time in the first weeks, all prostate patients are currently treated with RapidArc at our institute.

Aim of the present study is to report the technical and dosimetric aspects of the treatments as well as to summarize the acute toxicity findings.

Further investigations will aim to look at the long term clinical outcome and late toxicity in relation to dosimetric improvements in sparing of the organs at risk.

## Methods

Forty-five patients were treated with RapidArc (RA) from October 2008 to September 2009. Characteristics of patients are summarized in table 1. Most frequent stages were T1c and T2a-c (87% in total), N0 (96%) and all the patients were M0. Most of patients were non operated (96%) and the majority of them received hormonal therapy (69%). Gleason score was 6-7 in all cases. Median age was 72 years (range: 57-81 years).

**Table 1:** Summary of patients characteristics at treatment start.

Number of patients		45
**Age [years]** **(median and range)**		72 [57-81]

**Stage T**	**T1c**	22 (49%)
	**T2a-c**	17 (38%)
	**T3a-b**	6 (13%)

**Stage N**	**N0**	43 (96%)
	**N1**	1 (2%)
	**Nx**	1 (2%)

**Stage M**	**M0**	45 (100%)
	**M1**	0 (0%)

**Gleason score**	**6**	25 (56%)
	**7**	20 (44%)

**PSA at staging (μg/l)** **(Median and range)**		10.0 [4.7, 33.8]

**Hormonotherapy**	**Yes**	31 (69%)
	**No**	14 (31%)

**Surgery**	**Yes**	2 (4%)
	**No**	43 (96%)

Dose Prescription:		
Group A	**70+6 Gy**	7 (16%)
	**70+8 Gy**	9 (20%)
**Group B**	**78 Gy**	29 (64%)

The issue of target definition is highly debated for prostate cancer, particularly the inclusion of the seminal vesicles [14-18]. According to Kestin et al. [14] only 1% of low risk patients with PSA < 10 ng/mL or Gleason score < 6 and clinical stage ≤T2A, demonstrated seminal vesicles involvement. In their study, authors suggested to include only the proximal 2-2.5 cm of the vesicles for higher stages. Given the patient population of our study, this last strategy for clinical target volume definition was assumed as standard. Clinical Target Volume (CTV) was therefore defined as the prostate plus the basis of the seminal vesicles. Planning Target Volume (PTV) was the CTV with a margin of 1 cm in all directions except posteriorly where the margin was reduced to 0.5 cm. Some individualized reduction toward the rectum was applied whenever the rectum involvement was judged too high by the radiation oncologist.

Patients were divided into two groups: Group A (16 patients) received a total dose of 70Gy to a planning target volume (PTVII) including also the base of the seminal vesicles, plus a boost of 6-8 Gy to the prostate only (PTVI). Group B (29 patients, including two post operative patients) received a single course of treatment up to 78Gy to the entire PTV including prostate and base of seminal vesicles (or prostatic bed for patients who received surgery). In all cases, dose normalization was set to mean dose to PTV. In the framework of the initial phase of RapidArc clinical practice, no hypo-fractionation or dose escalation scheme was introduced and will be part of future investigations.

Organs at risk routinely considered in these patients are rectum, bladder, femoral heads and penile bulb. Rectum was delineated from 1 cm above anus to the sigma tract. In addition, as practice for all intensity modulated patients, the Healthy Tissue (HT) was defined as the patient's volume included in the CT dataset minus the PTV volume. No specific immobilisation systems were applied to prostate patients as well as no strong requirements on patient preparation. In this respect, patients were asked to empty bladder about half an hour prior to treatment and to regularize rectal evacuation during the first two weeks of treatment, also using small glycerine based enema one hour before treatment. Routine institutional image-based patient position verification protocols foresee 2D-2 D matching of orthogonal kV-MV images acquired with the On Board Imaging system installed at the accelerator with evaluation performed by radiographers and application of couch shifts if total vector length of displacement is smaller than 7 mm. Cone Beam CT is becoming part of our routine protocol and is now performed once a week in addition to the 2D-2 D matching (kV-MV) most common procedure. The introduction of RapidArc and a more systematic application of image-based patient position verification did not lead, in this first phase of clinical practice to any modification in target or margin definitions which were kept, for this group of patient, the same as for the previously adopted 3 D conformal technique.

RA plans were optimised for single arcs (rotation of 358°, from 179° to 181° CCW) for a Clinac 2100iX equipped with a Millennium-120 MLC (120 leaves with a resolution at isocentre of 5 mm for the inner 20 cm and 10 mm for the outer 2 × 10 cm) and a photon beam energy of 6MV. Further details on RA technique can be found in [4,5]. Plan optimisation was performed reinforcing, with appropriate dose volume constraints depending on the individual patient and not reported here, the achievement of the following planning objectives. For PTV plans were optimised aiming to obtain: V_95% _> 98% and V_107% _= 0.0%. Concerning bladder the aim was to keep mean dose < 45Gy and D_2% _< 80Gy. Planning objectives for rectum were: mean < 45Gy, V_50Gy _< 50%, V_60Gy _< 40%, V_70Gy _< 15%. For femoral heads, dose objective was D_2% _< 47Gy. The dose of 30Gy was considered as objective for mean dose to penile bulb. No explicit planning objectives were set for healthy tissue.

All dose distributions were computed with the Anisotropic Analytical Algorithm (AAA) implemented in the Eclipse planning system with a calculation grid resolution of 2.5 mm.

Technical features of treatments have been reported in terms of main delivery parameters (field and control point (CP) size, MU, MU/deg and MU/Gy, Dose Rate (DR), Gantry Speed (GS), Collimator angle, beam-on time). Results of pre-treatment plan quality assurance are reported as Gamma Agreement Index (GAI), i.e. the percentage of modulated field area passing the γ-index criteria of Low [19] with thresholds on dose difference set to ΔD = 3% of the significant maximum dose, and on Distance to Agreement set to DTA = 3 mm. Measurements and analysis were performed by means of the GLAaS methodology described in [20,21] based on absorbed dose to water from EPID measurements.

Dosimetric quality of treatments was measured from the dose volume histogram (DVH) analysis. For PTV the following data were reported: PTV coverage (D_2%_, D_98%_, V_95%_, V_107%_), homogeneity (D_5%_-D_95%_) and conformity (CI_90%_). CI_90% _is defined as the ratio between the volume of patient irradiated at 90% of the prescribed dose and the PTV volume. For OARs, the mean dose, the maximum dose (D_2%_) and appropriate values of V_xGy _(volume receiving at least × Gy) were scored. For Healthy Tissue, the integral dose DoseInt was reported as well. This is measured as the integral of the dose delivered to the entire HT and is expressed in Gy cm^3^.

Clinical outcome of treatments was recorded in terms of observed global acute toxicity, particularly dysuria, rectal toxicity and preservation of erectile function (in non operated patients). Toxicity scoring was assessed by non blind radiation oncologists in charge of the various patients and according to the National Cancer Institute Common Terminology Criteria of Adverse Effects scale (CTCAE version 3 [22]) as part of the routine visits during treatment and follow up protocols. Biochemical outcome was measured in terms of PSA reporting its value at treatment start and at end (6 weeks after RT) of radiation therapy course.

## Results

**Figure 1:**
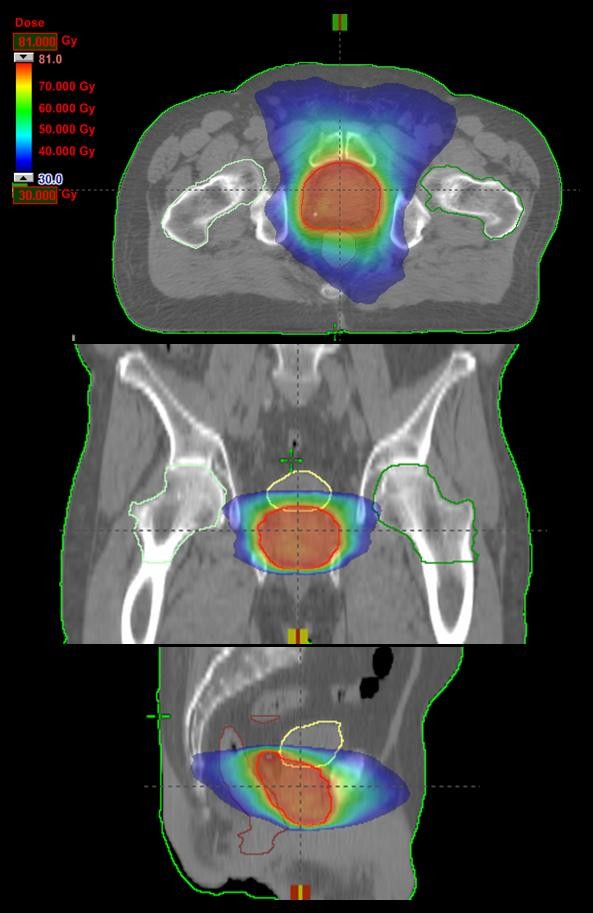
**Isodose distributions for one example patient for RA treatments for an axial plane, sagittal and coronal views**. Doses are shown in colorwash within the interval from 30 to 81 Gy.

**Figure 2:**
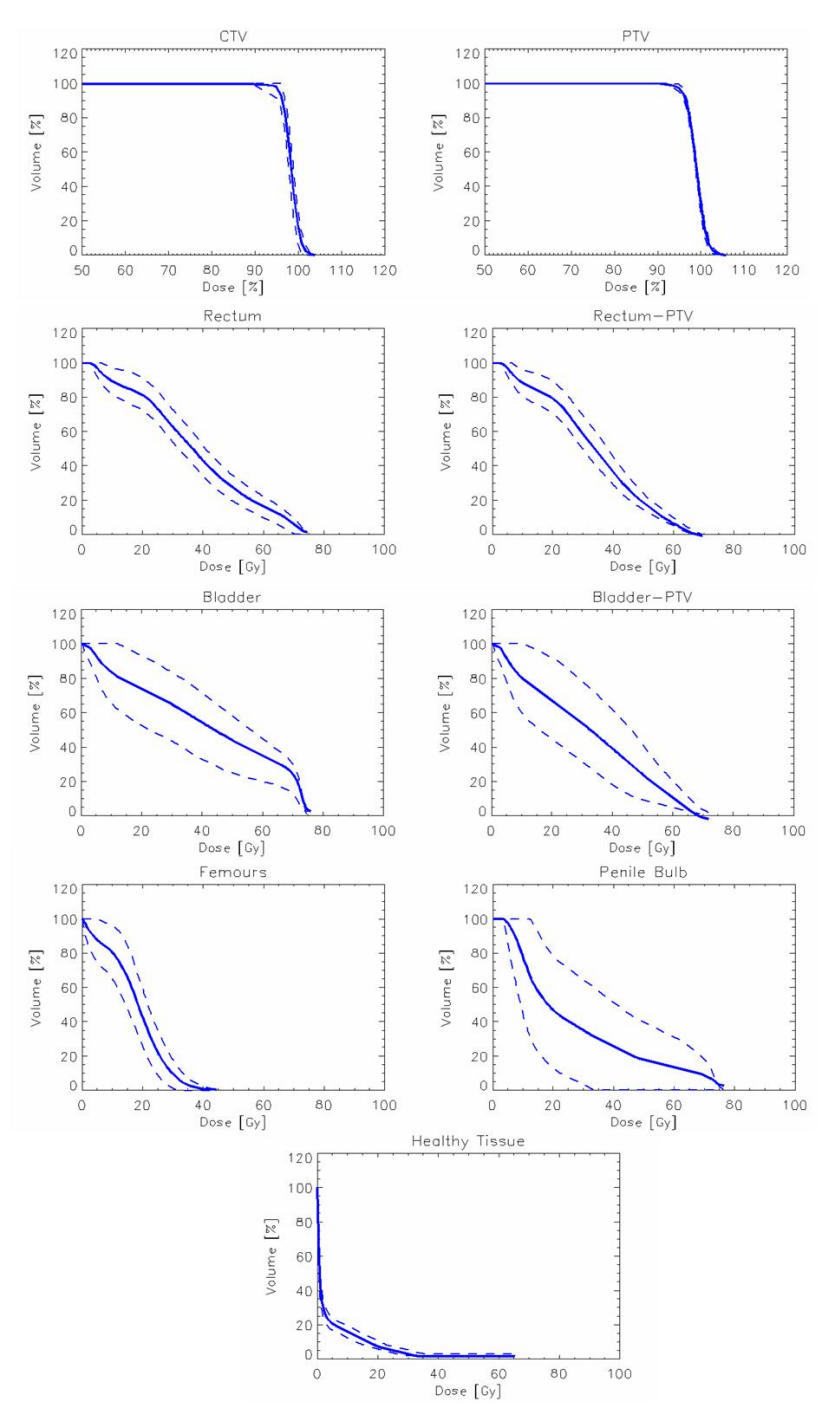
**Average Dose Volume Histograms for CTV, PTV, Bladder, Rectum, Femurs, Penile Bulb and Healthy Tissue for RA plans**. Dashed lines represent inter-patient variability at 1 standard deviation

Figure 1 shows examples of dose distributions for one patient in axial, coronal and sagittal planes. Colourwash is in the interval from 30 to 81Gy. Figure 2 reports the average DVHs (computed from all the 45 patients) for CTV, PTV, organs at risk and healthy tissue. Dashed lines represent the inter-patient variability at one standard deviation.

Table 2 summarises the technical features of the treatment characteristics. Table 3 and Table 4 report results of the DVH analysis. Table 5 records the clinical outcome of the treatments as early acute reactions and PSA values.

**Table 2:** Technical characteristics of RapidArc plans

	RA
**Number of arcs**	1
**Arc length [°]**	358 ± 0.0
**Beam energy**	6 MV (45/45)
**Beam on time [min]**	1.24 ± 0.0
**MU**	477 ± 73
**MU/Gy**	239 ± 37
**MU/deg**	1.3 ± 0.2
**Average Dose Rate [MU/min]**	383 ± 55
**Gantry speed [deg/sec]**	4.8 ± 0.0
**Collimator angle [°]**	24 ± 9
**Mean CP area [cm^2^]**	29 ± 6
**Mean field area [cm^2^]**	121 ± 25
**GAI [%]**	98.1 ± 1.1

MU: monitor units, CP: control point

**Table 3:** Summary of DVH analysis for CTVs and PTVs.

Parameter	Objectives	Group A	Group B
**CTV**			
**Volume [cm^3^]**	-	62.0 ± 25.6	67.0 ± 29.6
**Mean dose [%]**		99.5 ± 1.1	99.3 ± 0.5
**D_2% _[%]**	Minimise	102.9 ± 0.8	102.6 ± 1.0
**D_98% _[%]**	Maximise	96.3 ± 2.0	96.4 ± 0.5
**V_95% _[%]**	100%	97.1 ± 10.3	99.9 ± 0.2
**V_107% _[%]**	-	0.0 ± 0.0	0.0 ± 0.1
**D_5%_-D_95% _[%]**	Minimise	5.4 ± 1.7	5.1 ± 0.9

**PTVI (76-78 Gy)**			
**Volume [cm^3^]**	-	185.4 ± 68.2	245.0 ± 64.7
**Mean dose[%]**	100.0%	100.0 ± 0.0	100.0 ± 0.0
**D_2% _[%]**	Minimise	103.9 ± 0.9	104.1 ± 0.6
**D_98% _[%]**	> 95%	95.9 ± 0.8	95.4 ± 1.3
**V_95% _[%]**	> 98%	98.9 ± 0.9	98.2 ± 1.7
**V_107% _[%]**	0%	0.0 ± 0.1	0.1 ± 0.1
**D_5%_-D_95% _[%]**	Minimise	6.2 ± 1.0	6.7 ± 1.3
**CI_90%_**	1	1.3 ± 0.4	1.2 ± 0.1

**PTVII- PTVI (70Gy)**			
**Volume [cm^3^]**	-	47.5 ± 41.7	-
**Mean dose [%]**	-	101.3 ± 0.4	-
**D_2% _[%]**	Minimise	113.0 ± 1.4	-
**D_98% _[%]**	> 95%	96.9 ± 2.7	-
**V_95% _[%]**	> 98%	98.7 ± 1.3	-
**D_5%_-D_95% _[%]**	Minimise	12.7 ± 2.4	-
**CI_90%_**	1	1.4 ± 0.1	-

**Table 4:** Summary of DVH analysis for Rectum, Bladder, Femurs, Penile Bulb and Healhty Tissue.

Parameter	Objectives	All patients	All Patients
		**Rectum**	**Rectum-PTV**
**Volume [cm^3^]**	-	58.4 ± 17.9	53.6 ± 16.2
**Mean dose [Gy]**	< 45Gy	40.3 ± 4.2	36.7 ± 3.4
**V_50Gy _[%]**	< 60%	31.6 ± 7.6	24.7 ± 5.7
**V_60Gy _[%]**	< 45%	20.1 ± 6.6	12.1 ± 3.1
**V_70Gy _[%]**	< 25%	10.7 ± 5.5	2.4 ± 1.7
**NTCP [%]**	< 5%	2.7 ± 1.6	1.0 ± 0.4

		**Bladder**	**Bladder-PTV**
**Volume [cm^3^]**	-	151.0 ± 100.6	119.5 ± 93.5
**Mean dose [Gy]**	< 45Gy	44.5 ± 12.3	35.5 ± 11.1
**D_2% _[Gy]**	< 80Gy	79.4 ± 1.2	72.6 ± 2.4
**D_67% _[Gy]**	Minimize	29.4 ± 17.2	23.7 ± 14.0
**V_30Gy _[%]**	Minimize	65.8 ± 20.5	57.5 ± 22.7
**NTCP [%]**	< 5%	2.6 ± 4.0	0.1 ± 0.2

		**Femurs**	
**Volume [cm^3^]**	-	373.3 ± 86.7	
**Mean dose [Gy]**	Minimise	19.4 ± 4.2	
**V_45Gy _[%]**	Minimise	0.4 ± 1.0	
**D_2% _[Gy]**	< 47Gy	36.7 ± 5.4	

		**Penile Bulb**	
**Volume [cm^3^]**	-	3.3 ± 1.2	
**Mean dose [Gy]**	< 30Gy	28.5 ± 17.2	
**D_2% _[Gy]**	-	53.1 ± 23.7	

		**Healthy Tissue**	
**Volume [cm^3^]**	-	29054.2 ± 7186.1	
**Mean dose [Gy]**	Minimise	5.1 ± 1.0	
**V_10Gy _[%]**	Minimise	16.7 ± 3.2	
**V_20Gy _[%]**	Minimise	9.3 ± 2.3	
**V_30Gy _[%]**	Minimise	4.4 ± 1.2	
**DoseIntegral [10^5 ^Gy cm^3^]**	Minimise	1.45 ± 0.32.6	

**Table 5:** Clinical results at the end of radiotherapy.

Duration of RT [days]	Mean ± SD [range]	58 ± 4 [52-66]
**PSA pre-RT [μg/l]**	Median ± MAD [range]	6.7 ± 3.6 [0.1, 26.0]
**PSA post-RT [μg/l]**	Median ± MAD [range]	0.4 ± 0.4 [0.0, 6.8]

**Rectal acute toxicity**	G0	31/43 (72%)
	G1	12/43 (28%)
	G2	0/43(0%)
	G3	0/43(0%)

**Urinary acute toxicity** **(disuria)**	G0	8/42 (19%)
	G1	29/42 (69%)
	G2	5/42 (12%)
	G3	0/42 (0%)

**Erectile function**	Yes	14/41 (34%)
	Yes/No	4/41 (10%)
	No	23/41 (56%)

From the summary of main technical features it derives that treatment of prostate is characterised by relatively small field and control point areas resulting in a low output factor requiring high number of MU per minute and high average dose rate. With conventional fractionation and single arcs, gantry speed is kept constant at maximum speed.

Pre-treatment quality assurances of RA plans resulted in an average gamma agreement index GAI 3% superior to the acceptance threshold of 95% set as reference in our institute.

Dosimetric data showed that all planning objectives were met for PTVI and PTVII-PTVI (for group A only). Conformity of treatment, not explicitly considered as a planning objective, resulted acceptable. DVH analysis of organs at risk showed that all planning objectives were largely met when considering the fraction of organs not overlapping with PTV and when considering the entire organs (bladder and rectum) too.

Clinical data summarized in table 5 refer to acute and early results only scored at the end of treatment. All treatments were completed without unscheduled interruptions related to patients. Biochemical index PSA decreased to values close to zero at end of treatment (values are reported as median ± MAD (Median Absolute Deviation) and range). No severe (G2/3) acute rectal toxicity of any type was observed while 12% of patients experienced G2 dysuria (no events with higher grade). Erectile function was preserved in 44% of the patients.

## Discussion

Based on the results of an intensive program of pre-clinical investigations performed at planning level [3-9] to assess its reliability and potential benefit, RapidArc (a Volumetric Modulated Arc Therapy implemented on Varian linear accelerators and planning systems) was introduced in clinical practice of our institute since September 2008 for a variety of indications. The present study reports about the early findings from the treatment of a group of 45 patients affected by prostate carcinoma.

The main objective of this first phase of clinical introduction of RA is the assessment of the possibility to administer to patients standard radiotherapy treatments and moreover to investigate the potentials of improvements. These results were easily achieved in this group of patients: rectum tolerance, derived from [4,23] were respected with a reduction of a factor about 2 or more of the volume irradiated at medium-high doses in the range of 50-70 Gy and a mean reduction of about 5Gy for the mean dose. Tolerance on mean bladder dose, derived from [3], was in average met with a quite large inter-patient variability (seen also in the volume of the bladder itself) due to the absence of a strict bladder filling protocol in our institute. Penile bulb involvement resulted compatible with objectives enforced in other investigations [24].

The dosimetric results reported here might also support the activation of a second clinical phase, aiming to implement more aggressive fractionation schemes (either with hypo-fractionation or dose escalation approaches, and eventually including simultaneous integrated boost modalities to discriminate between prostate and seminal vesicles).

Having achieved the aimed quality of treatments, investigations of technical features of delivered plans, in comparison with previously reported data for different groups of patients [25], allow some general consideration.

Data reported in table 5 might be compared with the corresponding values from the pre-operative treatment of rectal patients reported in [25]. For prostate, the small volume and the relatively low modulation needed to fulfil the planning objectives, lead to an approximately three-fold smaller mean field area and about five-fold smaller average CP area. As a consequence the MU/Gy and average DR resulted significantly higher than in the case of rectum. These observations suggest that VMAT, in its RapidArc form, has an inherent site-specificity of the delivery parameters but that this is fully compensated by the flexibility of the optimisation engine to adapt to various modulation needs within the dynamic range of the free parameters (MLC and dose rate). Additional freedom would derive from gantry speed modulation in case of hypo-fractionation. Quality assurance measurements provided a confirmation of the robustness of the method and of the independence of treatment quality from the technical features. In fact, pre-treatment QA measurements lead to a gamma agreement index identical to what observed in the case of patients treated for rectal cancer [25]. This suggests also an invariance of quality between dose calculation and delivery for RapidArc treatments from crucial delivery parameters as average dose rate, field size, CP aperture and machine output (MU/Gy), despite their dependence from treatment site.

Concerning clinical workflow, delivery of about 1700 RA fractions to prostate, confirmed the significant reduction of effective treatment time anticipated in the preclinical phase [3-9]. For all patients and fractions, 1.24 minutes of beam on time were needed to deliver a single fraction with the exclusion of time needed to position the patients and to acquire data for image guidance. This shall be incremented by the time needed to perform image based patient position verification (depending from day to day and modality of imaging adopted). In total, with the procedures enforced in our institute, the average time needed to perform image based patient positioning, including evaluation and couch shifts is less than 4 minutes allowing a total time shorter than 10 minutes for a complete session.

The smoother process of RA could decrease the duration of the treatment reducing the risk of intra-fractional internal organ motion. In fact, bladder or rectum deformation was reported by several investigations. As an example, [26,27] using real time methods and electromagnetic tracking, showed a significant increase of prostate displacement with increasing treatment time (one eighth of patients showed displacements larger than 3 mm after 5 minutes from initial alignment increasing to one quarter after 10 minutes). According to these data and to treatment time recorded with RapidArc, it should therefore be possible to keep average displacements of prostate gland from position detected with pre-treatment imaging within acceptable levels (within 3 mm), allowing the possibility, if properly image guidance is performed, to eventually reduce CTV to PTV margins.

It is obvious that the present study cannot be considered as conclusive and that long term observation of patients is needed to measure outcome and late toxicity. These preliminary results are anyway encouraging further experience in this field.

## Conclusions

Forty-five patients with prostate carcinoma were treated with Volumetric Modulated Arc Therapy according to the RapidArc implementation in a clinical feasibility protocol. Quality of treatments resulted in an improvement of all planning objectives with regard to both target coverage and organs at risk sparing. Clinical outcome for early acute toxicity and assessment of biochemical outcome showed encouraging results. Future investigations will aim to appraise treatment of patients with inclusion of pelvic nodes and altered fractionation schemes. Long term outcome has to be evaluated with proper follow-up but the first phase achieved the primary goal to demonstrate safety and efficacy of RapidArc.

## Competing interests

LC acts as Scientific Advisor to Varian Medical Systems and is Head of Research and Technological Development to Oncology Institute of Southern Switzerland, IOSI, Bellinzona.

No special competing interest exists for any other author.

## Authors' contributions

GP, LC and AF coordinated the entire study. Patient accrual and clinical data collection was done by GP, AR, ES and MV. Data analysis, physics data and treatment planning data collection was conducted by AC, GN, EV and AF. The manuscript was prepared by LC. All authors read and approved the final manuscript.
